# 1,3,5-Tris(bromo­meth­yl)-2,4,6-trimeth­oxy­benzene

**DOI:** 10.1107/S1600536813008441

**Published:** 2013-04-10

**Authors:** Niklas Koch, Wilhelm Seichter, Monika Mazik

**Affiliations:** a Institut für Organische Chemie, Technische Universität Bergakademie Freiberg, Leipziger Strasse 29, D-09596 Freiberg/Sachsen, Germany

## Abstract

There are three independent mol­ecules in the asymmetric unit of the title compound, C_12_H_15_Br_3_O_3_, two of which have approximate trigonal symmetry, the third being conformationally different as it adopts near mirror symmetry. The crystal structure features C—H⋯Br inter­actions, a weak C—H⋯O hydrogen bond, π–π inter­actions [minimum ring centroid separation = 3.4927 (18) Å] and a short Br⋯Br contact [3.5894 (5) Å], resulting in a three-dimensional supramolecular network.

## Related literature

For the synthesis and sample applications of the title compound, see: Li *et al.* (2005 ▶); Kim *et al.* (2005 ▶); Holec *et al.* (2011 ▶); Simaan *et al.* (2003 ▶); Whiting & Hof (2012 ▶). For hydrogen bonds, see: Desiraju (2002 ▶); Steiner (2002 ▶). For halogen bonding, see: Awwadi *et al.* (2006 ▶); Metrangolo & Resnati (2008 ▶).
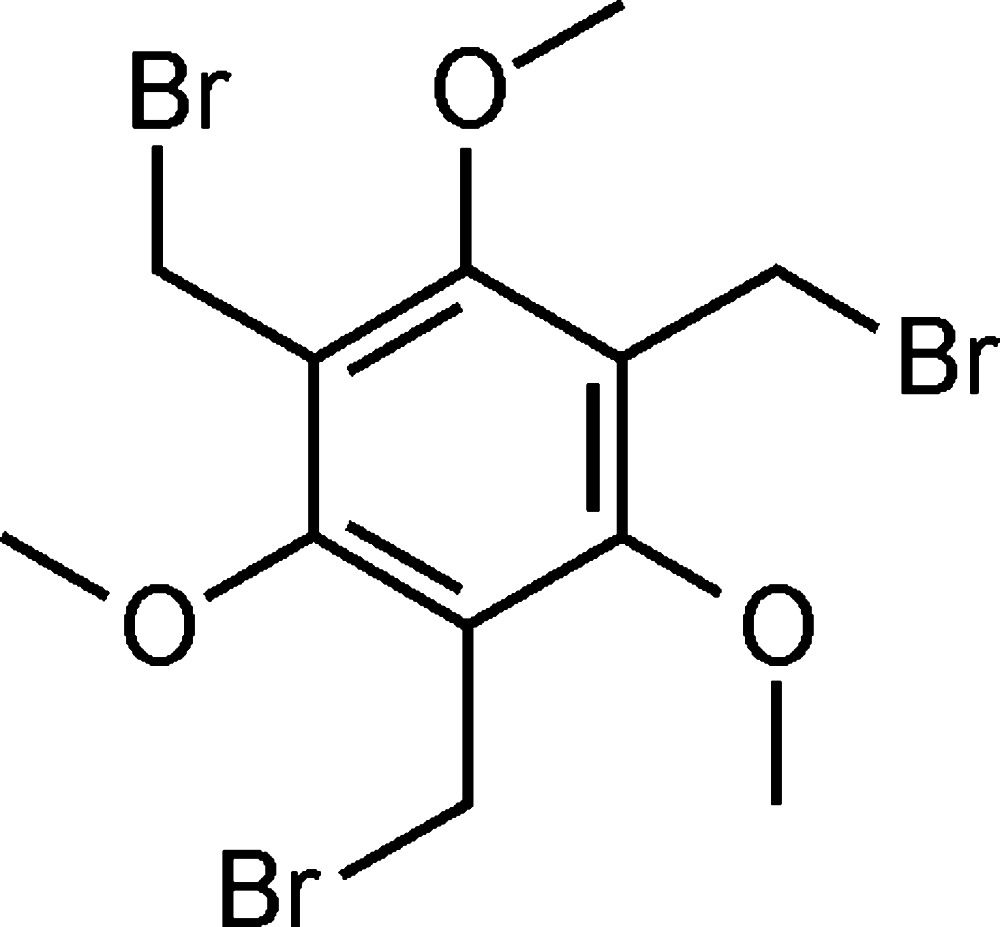


## Experimental

### 

#### Crystal data

C_12_H_15_Br_3_O_3_
*M* *_r_* = 446.94
Triclinic, 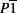

*a* = 9.7508 (5) Å
*b* = 15.0974 (7) Å
*c* = 16.5500 (7) Å
α = 114.433 (2)°
β = 92.903 (2)°
γ = 97.510 (2)°
*V* = 2184.29 (18) Å^3^
*Z* = 6
Mo *K*α radiation
μ = 8.31 mm^−1^
*T* = 100 K
0.55 × 0.44 × 0.27 mm

#### Data collection

Bruker APEXII CCD area-detector diffractometer
Absorption correction: multi-scan (*SADABS*; Bruker, 2008 ▶) *T*_min_ = 0.092, *T*_max_ = 0.213
44729 measured reflections
11474 independent reflections
9330 reflections with *I* > 2σ(*I*)
*R*_int_ = 0.034

#### Refinement

*R*[*F* ^2^ > 2σ(*F* ^2^)] = 0.030
*wR*(*F* ^2^) = 0.070
*S* = 1.02
11474 reflections
496 parameters
H-atom parameters constrained
Δρ_max_ = 1.17 e Å^−3^
Δρ_min_ = −1.10 e Å^−3^

### 

Data collection: *APEX2* (Bruker, 2008 ▶); cell refinement: *SAINT* (Bruker, 2008 ▶); data reduction: *SAINT*; program(s) used to solve structure: *SHELXS97* (Sheldrick, 2008 ▶); program(s) used to refine structure: *SHELXL97* (Sheldrick, 2008 ▶); molecular graphics: *ORTEP-3 for Windows* (Farrugia, 2012 ▶); software used to prepare material for publication: *SHELXTL* (Sheldrick, 2008 ▶).

## Supplementary Material

Crystal structure: contains datablock(s) I, global. DOI: 10.1107/S1600536813008441/zs2251sup1.cif

Structure factors: contains datablock(s) I. DOI: 10.1107/S1600536813008441/zs2251Isup2.hkl

Supplementary material file. DOI: 10.1107/S1600536813008441/zs2251Isup3.cml

Additional supplementary materials:  crystallographic information; 3D view; checkCIF report

## Figures and Tables

**Table 1:** Hydrogen-bond geometry (Å, °)

*D*—H⋯*A*	*D*—H	H⋯*A*	*D*⋯*A*	*D*—H⋯*A*
C8*A*—H8*A*1⋯Br1*A*	0.98	2.91	3.787 (3)	150
C8—H8*A*⋯Br1	0.98	2.84	3.702 (3)	148
C8—H8*C*⋯Br2	0.98	2.86	3.670 (3)	140
C7*B*—H7*B*2⋯Br3*B* ^i^	0.99	2.92	3.665 (3)	133
C10—H10*A*⋯Br2	0.98	2.85	3.717 (3)	149
C10—H10*C*⋯Br3	0.98	2.89	3.673 (3)	138
C10*A*—H10*D*⋯Br3*A*	0.98	2.78	3.659 (4)	149
C10*B*—H10*G*⋯Br2*B*	0.98	2.72	3.606 (3)	151
C10*B*—H10*I*⋯Br3*B*	0.98	2.86	3.663 (3)	140
C11—H11*B*⋯O1*A* ^ii^	0.99	2.55	3.429 (4)	148
C8*B*—H8*B*3⋯Br2*B*	0.98	2.85	3.697 (4)	146
C12*B*—H12*G*⋯Br3*B*	0.98	2.83	3.702 (3)	149

Symmetry codes: (i) 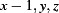; (ii) 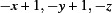.

**Fig. 1.**
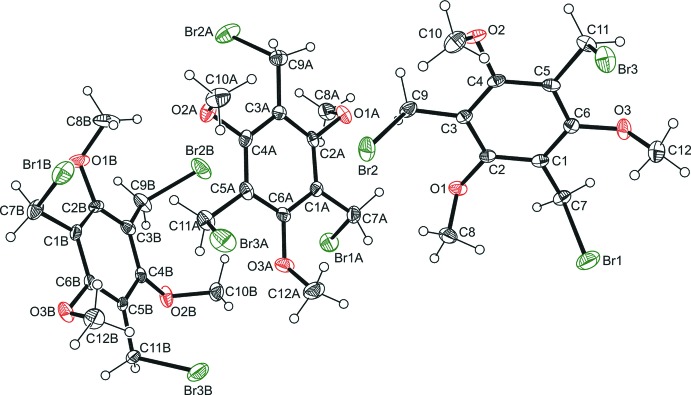
Atom numbering for the three independent molecules of the title compound in the asymmetric unit, showing 50% probability displacement ellipsoids for the non-H atoms.

**Fig. 2.**
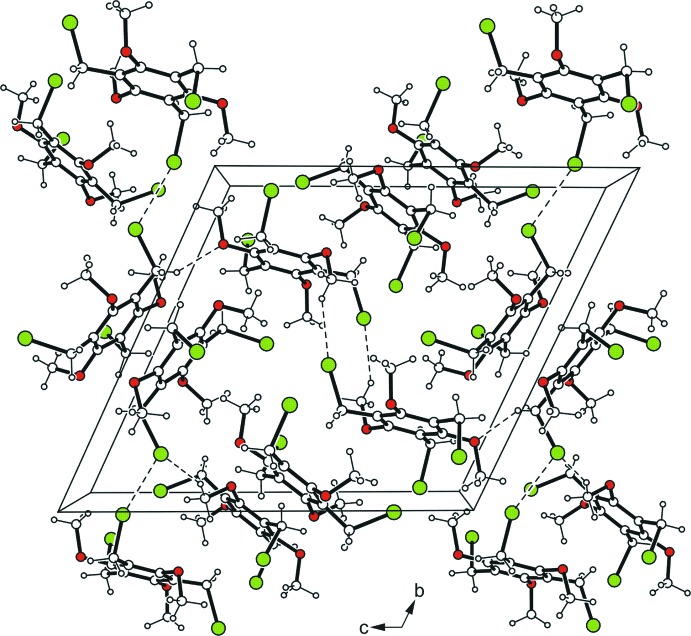
A packing diagram of the title compound viewed down the a axis. Intermolecular contacts are shown as dashed lines.

## supplementary crystallographic information

### Comment

1,3,5-Tris(bromomethyl)-2,4,6-trimethoxybenzene is widely used in modern synthetic chemistry, especially in supramolecular, macrocyclic and materials chemistry. For example, the trisubstituted 1,3,5-trimethoxybenzene scaffold finds application as a building block for organogelators (Li et al., 2005), macrocyclic cage compounds (Kim et al., 2005) and receptors for neutral or cationic guests (Holec et al., 2011; Whiting & Hof, 2012).

Our interest in the title compound, C_12_H_15_O_3_Br_3_, arises from its use in the synthesis of acyclic and macrocyclic receptors for biologically interesting species. This compound crystallizes in the space group P-1 with three independent molecules in the asymmetric unit (Fig. 1), two of which [the molecule with the C1–C6 ring and molecule B] adopt close to a trigonally symmetric conformation with all Br and methyl substituent groups oriented on one side of the aromatic ring (all cis). These molecules have a very approximate mirror symmetry (with atoms C7—C1···C4—O2 and C7B–C1B···C4B—O2B lying on the plane). The third conformational isomer (molecule A) can be described as 1,3-up, 5-down-tris(bromomethyl), 2-up, 4,6-down-trimethoxybenzene. Independent molecules participate in a different manner in intermolecular interactions. Pairs of inversion-related molecules with trigonal symmetry form face-to-face arene interactions with a minimum distance of 3.4927 (18) Å between the ring centroids. All molecules take part in the formation of C—H···Br hydrogen bonds [3.606 (3)–3.787 (3) Å, Table 1]. Moreover, the distance between the bromine atoms Br3 and Br1A [3.5894 (5) Å] and the well defined contact geometry [θ_1_ = 112.4°, θ_2_ = 155.1°] indicate the presence of a type II Br···Br interaction (Metrangolo & Resnati, 2008). The pattern of non-covalent intermolecular bonding is completed by a C11—H···O1A hydrogen bond [3.429 (4) Å].

### Experimental

The compound was prepared by a literature procedure with a slightly modified work-up (Li *et al.*, 2005). 1,3,5-trimethoxybenzene (500 mg, 2.97 mmol) and paraformaldehyde (330 mg, 10.99 mmol) were finely powdered and suspended in 5 ml of glacial acetic acid and stirred for 1 hour at room temperature, after which 3.5 ml of hydrogen bromide (30 wt % in HOAc) was added and the mixture stirred at 70 °C in a pressure tube for 3 hours. After cooling to room temperature the mixture was poured in 20 ml of destilled water and the resulting suspension was diluted with dichlormethane until a clear solution was obtained. After phase separation the aqueous layer was extracted twice with dichlormethane. The combined organic extracts were washed with water, dried over magnesium sulfate and concentrated in vacuo. The desired compound was obtained as a white solid after flash chromatography (SiO_2_, CH_2_Cl_2_ / hexane 7:1, *R_f_*═0.23) in 29 % yield (380 mg, 0.85 mmol). Analysis data: m.p. = 125 °C; ^1^H NMR (400 MHz, CDCl_3_) δ 4.14 (s, 9H, -OCH_3_), 4.60 (s, 6H, -CH_2_Br); ^13^C NMR (100 MHz, CDCl_3_) δ 22.45, 62.66, 123.29, 160.10; HR-ESI-MS (C_12_H_15_O_3_Br_3_): found 468.84501 [M+Na]^+^; calculated: 468.84434; Anal. Calcd (%) for C_12_H_15_O_3_Br_3_: C 32.25; H 3.38; found C 32.58; H 3.41. Suitable crystals of the title compound for X-ray analysis were obtained as colourless blocks by slow evaporation of a CDCl_3_ solution.

### Refinement

H atoms were positioned geometrically and allowed to ride on their respective parent atoms, with C—H = 0.98 Å and *U*_iso_(H) = 1.5 *U*_eq_(C) for methyl and C—H = 0.99 Å and *U*_iso_(H) = 1.2 *U*_eq_(C) for aryl methylene.

### Crystal data

**Table:**

C_12_H_15_Br_3_O_3_	*Z* = 6
*M**_r_* = 446.94	*F*(000) = 1296
Triclinic, *P*1	*D*_x_ = 2.039 Mg m^−^^3^
Hall symbol: -P 1	Melting point: 398 K
*a* = 9.7508 (5) Å	Mo *K*α radiation, λ = 0.71073 Å
*b* = 15.0974 (7) Å	Cell parameters from 9450 reflections
*c* = 16.5500 (7) Å	θ = 2.4–29.2°
α = 114.433 (2)°	µ = 8.31 mm^−^^1^
β = 92.903 (2)°	*T* = 100 K
γ = 97.510 (2)°	Block, colourless
*V* = 2184.29 (18) Å^3^	0.55 × 0.44 × 0.27 mm

### Data collection

**Table:**

Bruker APEXII CCD area-detector diffractometer	11474 independent reflections
Radiation source: fine-focus sealed tube	9330 reflections with *I* > 2σ(*I*)
Graphite monochromator	*R*_int_ = 0.034
φ and ω scans	θ_max_ = 28.9°, θ_min_ = 1.5°
Absorption correction: multi-scan (*SADABS*; Bruker, 2008)	*h* = −13→13
*T*_min_ = 0.092, *T*_max_ = 0.213	*k* = −20→20
44729 measured reflections	*l* = −22→21

### Refinement

**Table:**

Refinement on *F*^2^	Primary atom site location: structure-invariant direct methods
Least-squares matrix: full	Secondary atom site location: difference Fourier map
*R*[*F*^2^ > 2σ(*F*^2^)] = 0.030	Hydrogen site location: inferred from neighbouring sites
*wR*(*F*^2^) = 0.070	H-atom parameters constrained
*S* = 1.02	*w* = 1/[σ^2^(*F*_o_^2^) + (0.0264*P*)^2^ + 3.7624*P*] where *P* = (*F*_o_^2^ + 2*F*_c_^2^)/3
11474 reflections	(Δ/σ)_max_ < 0.001
496 parameters	Δρ_max_ = 1.17 e Å^−^^3^
0 restraints	Δρ_min_ = −1.10 e Å^−^^3^

### Special details

**Table:**

Geometry. All e.s.d.'s (except the e.s.d. in the dihedral angle between two l.s. planes) are estimated using the full covariance matrix. The cell e.s.d.'s are taken into account individually in the estimation of e.s.d.'s in distances, angles and torsion angles; correlations between e.s.d.'s in cell parameters are only used when they are defined by crystal symmetry. An approximate (isotropic) treatment of cell e.s.d.'s is used for estimating e.s.d.'s involving l.s. planes.
Refinement. Refinement of *F*^2^ against ALL reflections. The weighted *R*-factor *wR* and goodness of fit *S* are based on *F*^2^, conventional *R*-factors *R* are based on *F*, with *F* set to zero for negative *F*^2^. The threshold expression of *F*^2^ > σ(*F*^2^) is used only for calculating *R*-factors(gt) *etc*. and is not relevant to the choice of reflections for refinement. *R*-factors based on *F*^2^ are statistically about twice as large as those based on *F*, and *R*- factors based on ALL data will be even larger.

### Fractional atomic coordinates and isotropic or equivalent isotropic displacement parameters (Å^2^)

**Table:**

	*x*	*y*	*z*	*U*_iso_*/*U*_eq_	
Br1	1.00621 (3)	0.53065 (2)	0.15704 (2)	0.01897 (7)	
Br2	0.37963 (3)	0.50849 (2)	0.298180 (19)	0.02310 (7)	
Br3	0.62290 (4)	0.84512 (2)	0.16701 (2)	0.02290 (7)	
O1	0.6269 (2)	0.39201 (14)	0.14146 (13)	0.0159 (4)	
O2	0.3537 (2)	0.62713 (15)	0.12679 (13)	0.0175 (4)	
O3	0.8075 (2)	0.62276 (15)	0.03078 (13)	0.0175 (4)	
C1	0.7213 (3)	0.51009 (19)	0.08885 (16)	0.0117 (5)	
C2	0.6141 (3)	0.47348 (19)	0.12529 (17)	0.0116 (5)	
C3	0.4904 (3)	0.51168 (19)	0.13990 (17)	0.0114 (5)	
C4	0.4777 (3)	0.5919 (2)	0.12020 (17)	0.0126 (5)	
C5	0.5847 (3)	0.63304 (19)	0.08661 (17)	0.0119 (5)	
C6	0.7045 (3)	0.5906 (2)	0.07095 (17)	0.0125 (5)	
C7	0.8445 (3)	0.4616 (2)	0.06466 (18)	0.0149 (6)	
H7A	0.8692	0.4604	0.0070	0.018*	
H7B	0.8217	0.3925	0.0569	0.018*	
C8	0.7034 (3)	0.4162 (2)	0.22684 (19)	0.0184 (6)	
H8A	0.7886	0.4629	0.2354	0.028*	
H8B	0.7278	0.3560	0.2285	0.028*	
H8C	0.6457	0.4461	0.2745	0.028*	
C9	0.3726 (3)	0.4622 (2)	0.16750 (18)	0.0158 (6)	
H9A	0.3728	0.3902	0.1399	0.019*	
H9B	0.2841	0.4738	0.1445	0.019*	
C10	0.3348 (3)	0.6978 (2)	0.2145 (2)	0.0220 (6)	
H10A	0.3246	0.6650	0.2545	0.033*	
H10B	0.2510	0.7260	0.2108	0.033*	
H10C	0.4160	0.7506	0.2378	0.033*	
C11	0.5653 (3)	0.7161 (2)	0.06288 (18)	0.0170 (6)	
H11A	0.4661	0.7091	0.0414	0.020*	
H11B	0.6211	0.7133	0.0138	0.020*	
C12	0.9015 (3)	0.7114 (2)	0.0875 (2)	0.0199 (6)	
H12A	0.8698	0.7681	0.0826	0.030*	
H12B	0.9951	0.7066	0.0690	0.030*	
H12C	0.9035	0.7200	0.1496	0.030*	
Br1A	0.49210 (3)	0.04165 (2)	0.135857 (19)	0.01964 (7)	
Br2A	−0.15090 (4)	0.16662 (2)	0.10600 (3)	0.03205 (9)	
Br3A	0.30371 (4)	0.42815 (2)	0.51703 (2)	0.02858 (8)	
O1A	0.2461 (2)	0.19509 (14)	0.06502 (12)	0.0166 (4)	
O2A	0.0067 (2)	0.31567 (15)	0.32249 (14)	0.0187 (4)	
O3A	0.4567 (2)	0.22737 (15)	0.34440 (14)	0.0190 (4)	
C1A	0.3562 (3)	0.21360 (19)	0.20460 (18)	0.0127 (5)	
C2A	0.2423 (3)	0.22089 (19)	0.15496 (17)	0.0121 (5)	
C3A	0.1271 (3)	0.25851 (19)	0.19418 (17)	0.0123 (5)	
C4A	0.1243 (3)	0.28472 (19)	0.28527 (18)	0.0135 (5)	
C5A	0.2333 (3)	0.27421 (19)	0.33681 (18)	0.0142 (6)	
C6A	0.3492 (3)	0.24041 (19)	0.29556 (18)	0.0131 (5)	
C7A	0.4828 (3)	0.1800 (2)	0.1627 (2)	0.0176 (6)	
H7A1	0.5665	0.2222	0.2035	0.021*	
H7A2	0.4844	0.1886	0.1066	0.021*	
C8A	0.1973 (3)	0.0915 (2)	0.01111 (18)	0.0196 (6)	
H8A1	0.2501	0.0526	0.0322	0.029*	
H8A2	0.2105	0.0756	−0.0514	0.029*	
H8A3	0.0982	0.0757	0.0159	0.029*	
C9A	0.0112 (3)	0.2722 (2)	0.14060 (19)	0.0176 (6)	
H9A1	0.0453	0.2743	0.0860	0.021*	
H9A2	−0.0180	0.3363	0.1758	0.021*	
C10A	0.0133 (4)	0.4206 (2)	0.3641 (2)	0.0234 (7)	
H10D	0.0850	0.4497	0.4157	0.035*	
H10E	−0.0772	0.4360	0.3840	0.035*	
H10F	0.0364	0.4477	0.3210	0.035*	
C11A	0.2225 (3)	0.2920 (2)	0.43150 (18)	0.0185 (6)	
H11C	0.2709	0.2451	0.4449	0.022*	
H11D	0.1232	0.2789	0.4395	0.022*	
C12A	0.5650 (3)	0.3111 (2)	0.3896 (2)	0.0247 (7)	
H12D	0.6494	0.2991	0.3598	0.037*	
H12E	0.5845	0.3216	0.4518	0.037*	
H12F	0.5348	0.3698	0.3879	0.037*	
Br1B	−0.11391 (3)	0.31688 (2)	0.56329 (2)	0.02455 (7)	
Br2B	0.06858 (4)	−0.00924 (3)	0.18891 (2)	0.03241 (9)	
Br3B	0.51099 (3)	0.18450 (2)	0.54894 (2)	0.01948 (7)	
O1B	−0.1658 (2)	0.05465 (17)	0.37834 (16)	0.0261 (5)	
O2B	0.2856 (2)	−0.03484 (15)	0.37591 (14)	0.0199 (5)	
O3B	0.1524 (2)	0.19733 (15)	0.64356 (12)	0.0183 (4)	
C1B	−0.0038 (3)	0.1345 (2)	0.50967 (18)	0.0134 (5)	
C2B	−0.0329 (3)	0.0707 (2)	0.41889 (19)	0.0147 (6)	
C3B	0.0642 (3)	0.0156 (2)	0.37157 (18)	0.0144 (6)	
C4B	0.1936 (3)	0.02553 (19)	0.41748 (17)	0.0114 (5)	
C5B	0.2266 (3)	0.08767 (19)	0.50829 (17)	0.0109 (5)	
C6B	0.1270 (3)	0.14209 (19)	0.55285 (17)	0.0113 (5)	
C7B	−0.1125 (3)	0.1851 (2)	0.5607 (2)	0.0206 (6)	
H7B1	−0.0971	0.1921	0.6228	0.025*	
H7B2	−0.2048	0.1438	0.5338	0.025*	
C8B	−0.1914 (4)	0.1133 (3)	0.3320 (2)	0.0293 (8)	
H8B1	−0.1553	0.1828	0.3706	0.044*	
H8B2	−0.2919	0.1051	0.3159	0.044*	
H8B3	−0.1447	0.0922	0.2777	0.044*	
C9B	0.0257 (4)	−0.0599 (2)	0.27820 (19)	0.0217 (7)	
H9B1	−0.0753	−0.0855	0.2693	0.026*	
H9B2	0.0761	−0.1156	0.2686	0.026*	
C10B	0.3755 (3)	−0.0024 (2)	0.3238 (2)	0.0194 (6)	
H10G	0.3190	0.0055	0.2773	0.029*	
H10H	0.4353	−0.0515	0.2958	0.029*	
H10I	0.4335	0.0609	0.3626	0.029*	
C11B	0.3590 (3)	0.0895 (2)	0.55699 (19)	0.0152 (6)	
H11E	0.3846	0.0227	0.5317	0.018*	
H11F	0.3465	0.1077	0.6206	0.018*	
C12B	0.2247 (3)	0.2960 (2)	0.67127 (19)	0.0209 (6)	
H12G	0.3122	0.2942	0.6444	0.031*	
H12H	0.2448	0.3279	0.7365	0.031*	
H12I	0.1665	0.3334	0.6518	0.031*	

### Atomic displacement parameters (Å^2^)

**Table:**

	*U*^11^	*U*^22^	*U*^33^	*U*^12^	*U*^13^	*U*^23^
Br1	0.00939 (14)	0.02295 (15)	0.02450 (15)	0.00310 (11)	−0.00017 (11)	0.01013 (12)
Br2	0.02417 (17)	0.02506 (16)	0.01819 (14)	0.00046 (13)	0.00854 (12)	0.00773 (12)
Br3	0.03147 (19)	0.01265 (14)	0.02433 (15)	0.00570 (12)	0.01059 (13)	0.00620 (12)
O1	0.0178 (11)	0.0117 (9)	0.0171 (10)	0.0015 (8)	−0.0034 (8)	0.0060 (8)
O2	0.0115 (10)	0.0208 (10)	0.0169 (10)	0.0074 (8)	−0.0018 (8)	0.0036 (8)
O3	0.0198 (11)	0.0181 (10)	0.0131 (9)	−0.0012 (8)	0.0051 (8)	0.0062 (8)
C1	0.0125 (14)	0.0108 (12)	0.0069 (11)	0.0011 (10)	−0.0031 (10)	−0.0003 (10)
C2	0.0117 (14)	0.0109 (12)	0.0092 (12)	0.0011 (10)	−0.0023 (10)	0.0021 (10)
C3	0.0103 (13)	0.0113 (12)	0.0083 (11)	−0.0005 (10)	−0.0026 (10)	0.0010 (10)
C4	0.0102 (14)	0.0156 (13)	0.0080 (11)	0.0029 (11)	−0.0022 (10)	0.0012 (10)
C5	0.0141 (14)	0.0113 (12)	0.0075 (11)	0.0029 (10)	−0.0027 (10)	0.0013 (10)
C6	0.0132 (14)	0.0143 (13)	0.0066 (11)	0.0003 (11)	−0.0007 (10)	0.0020 (10)
C7	0.0120 (14)	0.0167 (14)	0.0123 (12)	0.0034 (11)	0.0005 (11)	0.0021 (11)
C8	0.0199 (16)	0.0198 (15)	0.0185 (14)	0.0040 (12)	−0.0001 (12)	0.0112 (12)
C9	0.0115 (14)	0.0187 (14)	0.0129 (12)	0.0001 (11)	−0.0013 (11)	0.0035 (11)
C10	0.0176 (16)	0.0213 (15)	0.0224 (15)	0.0057 (13)	0.0048 (13)	0.0038 (12)
C11	0.0217 (16)	0.0168 (14)	0.0146 (13)	0.0059 (12)	0.0006 (12)	0.0082 (11)
C12	0.0196 (16)	0.0169 (14)	0.0225 (15)	0.0004 (12)	0.0047 (13)	0.0083 (12)
Br1A	0.02175 (16)	0.01827 (14)	0.02136 (14)	0.00848 (12)	0.00611 (12)	0.00893 (12)
Br2A	0.01615 (17)	0.02113 (16)	0.0444 (2)	0.00290 (13)	−0.00690 (14)	0.00098 (14)
Br3A	0.0378 (2)	0.02262 (16)	0.01586 (14)	−0.00243 (14)	−0.00652 (13)	0.00207 (12)
O1A	0.0254 (12)	0.0141 (10)	0.0123 (9)	0.0040 (8)	0.0052 (8)	0.0070 (8)
O2A	0.0153 (11)	0.0169 (10)	0.0224 (10)	0.0039 (8)	0.0077 (9)	0.0061 (8)
O3A	0.0194 (11)	0.0167 (10)	0.0228 (10)	0.0000 (9)	−0.0052 (9)	0.0119 (9)
C1A	0.0129 (14)	0.0090 (12)	0.0182 (13)	0.0013 (10)	0.0031 (11)	0.0077 (10)
C2A	0.0164 (15)	0.0090 (12)	0.0112 (12)	−0.0005 (10)	0.0029 (11)	0.0052 (10)
C3A	0.0121 (14)	0.0096 (12)	0.0144 (12)	−0.0016 (10)	−0.0010 (11)	0.0055 (10)
C4A	0.0133 (14)	0.0097 (12)	0.0179 (13)	0.0013 (11)	0.0049 (11)	0.0060 (10)
C5A	0.0194 (15)	0.0090 (12)	0.0128 (12)	−0.0022 (11)	0.0018 (11)	0.0045 (10)
C6A	0.0149 (14)	0.0097 (12)	0.0162 (13)	−0.0014 (11)	−0.0005 (11)	0.0081 (11)
C7A	0.0157 (15)	0.0175 (14)	0.0231 (14)	0.0019 (12)	0.0052 (12)	0.0121 (12)
C8A	0.0232 (17)	0.0195 (15)	0.0120 (13)	0.0013 (13)	−0.0006 (12)	0.0038 (11)
C9A	0.0174 (15)	0.0156 (14)	0.0191 (14)	0.0020 (12)	−0.0011 (12)	0.0073 (11)
C10A	0.0230 (18)	0.0214 (16)	0.0205 (15)	0.0105 (13)	0.0019 (13)	0.0017 (12)
C11A	0.0242 (17)	0.0133 (13)	0.0161 (13)	−0.0016 (12)	0.0007 (12)	0.0058 (11)
C12A	0.0166 (16)	0.0233 (16)	0.0326 (17)	−0.0037 (13)	−0.0067 (13)	0.0135 (14)
Br1B	0.02098 (17)	0.01649 (14)	0.03671 (17)	0.00742 (12)	0.00790 (14)	0.01007 (13)
Br2B	0.0393 (2)	0.03877 (19)	0.01701 (15)	−0.01025 (16)	−0.00771 (14)	0.01582 (14)
Br3B	0.00936 (14)	0.02310 (15)	0.02693 (15)	−0.00075 (11)	−0.00188 (11)	0.01300 (12)
O1B	0.0120 (11)	0.0310 (12)	0.0411 (13)	−0.0071 (9)	−0.0111 (10)	0.0258 (11)
O2B	0.0269 (12)	0.0170 (10)	0.0243 (11)	0.0126 (9)	0.0171 (9)	0.0129 (9)
O3B	0.0257 (12)	0.0177 (10)	0.0100 (9)	0.0003 (9)	0.0035 (8)	0.0053 (8)
C1B	0.0100 (14)	0.0147 (13)	0.0210 (13)	0.0033 (11)	0.0060 (11)	0.0122 (11)
C2B	0.0080 (13)	0.0159 (13)	0.0226 (14)	−0.0051 (11)	−0.0049 (11)	0.0132 (12)
C3B	0.0177 (15)	0.0120 (13)	0.0138 (12)	−0.0024 (11)	−0.0015 (11)	0.0076 (11)
C4B	0.0138 (14)	0.0100 (12)	0.0138 (12)	0.0031 (10)	0.0057 (11)	0.0076 (10)
C5B	0.0090 (13)	0.0123 (12)	0.0141 (12)	0.0005 (10)	0.0029 (10)	0.0084 (10)
C6B	0.0134 (14)	0.0110 (12)	0.0102 (12)	0.0002 (10)	0.0028 (10)	0.0054 (10)
C7B	0.0159 (16)	0.0193 (15)	0.0349 (17)	0.0088 (12)	0.0109 (13)	0.0172 (13)
C8B	0.0264 (19)	0.0271 (17)	0.0370 (19)	0.0033 (14)	−0.0135 (15)	0.0185 (15)
C9B	0.0324 (19)	0.0172 (14)	0.0141 (13)	−0.0042 (13)	−0.0025 (13)	0.0082 (12)
C10B	0.0194 (16)	0.0219 (15)	0.0196 (14)	0.0071 (12)	0.0093 (12)	0.0098 (12)
C11B	0.0094 (14)	0.0196 (14)	0.0203 (14)	0.0007 (11)	0.0004 (11)	0.0128 (12)
C12B	0.0219 (17)	0.0184 (15)	0.0141 (13)	−0.0004 (12)	−0.0008 (12)	0.0002 (11)

### Geometric parameters (Å, º)

**Table:**

Br1—C7	1.979 (3)	C3A—C9A	1.488 (4)
Br2—C9	1.973 (3)	C7A—H7A1	0.9900
Br3—C11	1.980 (3)	C7A—H7A2	0.9900
O1—C2	1.382 (3)	C8A—H8A1	0.9800
O1—C8	1.443 (3)	C8A—H8A2	0.9800
O2—C4	1.374 (3)	C8A—H8A3	0.9800
O2—C10	1.441 (3)	C9A—H9A1	0.9900
O3—C6	1.376 (3)	C9A—H9A2	0.9900
O3—C12	1.437 (4)	C10A—H10D	0.9800
C1—C6	1.393 (4)	C10A—H10E	0.9800
C1—C2	1.397 (4)	C10A—H10F	0.9800
C1—C7	1.475 (4)	C11A—H11C	0.9900
C2—C3	1.393 (4)	C11A—H11D	0.9900
C3—C4	1.397 (4)	C12A—H12D	0.9800
C3—C9	1.483 (4)	C12A—H12E	0.9800
C4—C5	1.399 (4)	C12A—H12F	0.9800
C5—C6	1.390 (4)	Br1B—C7B	1.974 (3)
C5—C11	1.491 (4)	Br2B—C9B	1.964 (3)
C7—H7A	0.9900	Br3B—C11B	1.973 (3)
C7—H7B	0.9900	O1B—C2B	1.373 (3)
C8—H8A	0.9800	O1B—C8B	1.425 (4)
C8—H8B	0.9800	O2B—C4B	1.366 (3)
C8—H8C	0.9800	O2B—C10B	1.437 (3)
C9—H9A	0.9900	O3B—C6B	1.372 (3)
C9—H9B	0.9900	O3B—C12B	1.433 (4)
C10—H10A	0.9800	C1B—C6B	1.397 (4)
C10—H10B	0.9800	C1B—C2B	1.398 (4)
C10—H10C	0.9800	C1B—C7B	1.480 (4)
C11—H11A	0.9900	C2B—C3B	1.394 (4)
C11—H11B	0.9900	C3B—C4B	1.397 (4)
C12—H12A	0.9800	C3B—C9B	1.483 (4)
C12—H12B	0.9800	C4B—C5B	1.396 (4)
C12—H12C	0.9800	C5B—C6B	1.394 (4)
Br1A—C7A	1.963 (3)	C5B—C11B	1.478 (4)
Br3A—C11A	1.977 (3)	C7B—H7B1	0.9900
Br2A—C9A	1.965 (3)	C7B—H7B2	0.9900
O3A—C6A	1.375 (3)	C8B—H8B1	0.9800
O3A—C12A	1.435 (4)	C8B—H8B2	0.9800
O2A—C4A	1.373 (3)	C8B—H8B3	0.9800
O2A—C10A	1.433 (4)	C9B—H9B1	0.9900
O1A—C2A	1.378 (3)	C9B—H9B2	0.9900
O1A—C8A	1.441 (3)	C10B—H10G	0.9800
C1A—C2A	1.391 (4)	C10B—H10H	0.9800
C1A—C6A	1.396 (4)	C10B—H10I	0.9800
C1A—C7A	1.490 (4)	C11B—H11E	0.9900
C6A—C5A	1.394 (4)	C11B—H11F	0.9900
C5A—C4A	1.395 (4)	C12B—H12G	0.9800
C5A—C11A	1.488 (4)	C12B—H12H	0.9800
C4A—C3A	1.394 (4)	C12B—H12I	0.9800
C3A—C2A	1.394 (4)		
			
C2—O1—C8	113.5 (2)	H12D—C12A—H12E	109.5
C4—O2—C10	115.5 (2)	O3A—C12A—H12F	109.5
C6—O3—C12	116.2 (2)	H12D—C12A—H12F	109.5
C6—C1—C2	117.8 (3)	H12E—C12A—H12F	109.5
C6—C1—C7	120.7 (2)	C5A—C11A—Br3A	112.86 (18)
C2—C1—C7	121.4 (2)	C5A—C11A—H11C	109.0
O1—C2—C3	118.7 (2)	Br3A—C11A—H11C	109.0
O1—C2—C1	118.8 (2)	C5A—C11A—H11D	109.0
C3—C2—C1	122.3 (2)	Br3A—C11A—H11D	109.0
C2—C3—C4	117.8 (2)	H11C—C11A—H11D	107.8
C2—C3—C9	120.3 (2)	O2A—C10A—H10D	109.5
C4—C3—C9	121.6 (3)	O2A—C10A—H10E	109.5
O2—C4—C3	119.9 (2)	H10D—C10A—H10E	109.5
O2—C4—C5	118.3 (2)	O2A—C10A—H10F	109.5
C3—C4—C5	121.6 (3)	H10D—C10A—H10F	109.5
C6—C5—C4	118.4 (2)	H10E—C10A—H10F	109.5
C6—C5—C11	121.3 (2)	C3A—C9A—Br2A	112.80 (19)
C4—C5—C11	120.2 (3)	C3A—C9A—H9A1	109.0
O3—C6—C5	120.6 (2)	Br2A—C9A—H9A1	109.0
O3—C6—C1	117.2 (2)	C3A—C9A—H9A2	109.0
C5—C6—C1	122.0 (2)	Br2A—C9A—H9A2	109.0
C1—C7—Br1	111.74 (18)	H9A1—C9A—H9A2	107.8
C1—C7—H7A	109.3	O1A—C8A—H8A1	109.5
Br1—C7—H7A	109.3	O1A—C8A—H8A2	109.5
C1—C7—H7B	109.3	H8A1—C8A—H8A2	109.5
Br1—C7—H7B	109.3	O1A—C8A—H8A3	109.5
H7A—C7—H7B	107.9	H8A1—C8A—H8A3	109.5
O1—C8—H8A	109.5	H8A2—C8A—H8A3	109.5
O1—C8—H8B	109.5	C2B—O1B—C8B	116.6 (2)
H8A—C8—H8B	109.5	C4B—O2B—C10B	116.1 (2)
O1—C8—H8C	109.5	C6B—O3B—C12B	115.2 (2)
H8A—C8—H8C	109.5	C6B—C1B—C2B	118.0 (3)
H8B—C8—H8C	109.5	C6B—C1B—C7B	120.6 (3)
C3—C9—Br2	113.33 (19)	C2B—C1B—C7B	121.1 (3)
C3—C9—H9A	108.9	O1B—C2B—C3B	119.4 (3)
Br2—C9—H9A	108.9	O1B—C2B—C1B	118.3 (3)
C3—C9—H9B	108.9	C3B—C2B—C1B	121.9 (3)
Br2—C9—H9B	108.9	C2B—C3B—C4B	118.1 (2)
H9A—C9—H9B	107.7	C2B—C3B—C9B	120.9 (3)
O2—C10—H10A	109.5	C4B—C3B—C9B	120.6 (3)
O2—C10—H10B	109.5	O2B—C4B—C5B	118.0 (2)
H10A—C10—H10B	109.5	O2B—C4B—C3B	119.7 (2)
O2—C10—H10C	109.5	C5B—C4B—C3B	121.9 (3)
H10A—C10—H10C	109.5	C6B—C5B—C4B	118.1 (3)
H10B—C10—H10C	109.5	C6B—C5B—C11B	121.1 (2)
C5—C11—Br3	111.17 (18)	C4B—C5B—C11B	120.7 (2)
C5—C11—H11A	109.4	O3B—C6B—C5B	119.1 (2)
Br3—C11—H11A	109.4	O3B—C6B—C1B	118.6 (2)
C5—C11—H11B	109.4	C5B—C6B—C1B	122.0 (2)
Br3—C11—H11B	109.4	C1B—C7B—Br1B	113.00 (19)
H11A—C11—H11B	108.0	C1B—C7B—H7B1	109.0
O3—C12—H12A	109.5	Br1B—C7B—H7B1	109.0
O3—C12—H12B	109.5	C1B—C7B—H7B2	109.0
H12A—C12—H12B	109.5	Br1B—C7B—H7B2	109.0
O3—C12—H12C	109.5	H7B1—C7B—H7B2	107.8
H12A—C12—H12C	109.5	O1B—C8B—H8B1	109.5
H12B—C12—H12C	109.5	O1B—C8B—H8B2	109.5
C6A—O3A—C12A	116.2 (2)	H8B1—C8B—H8B2	109.5
C4A—O2A—C10A	114.8 (2)	O1B—C8B—H8B3	109.5
C2A—O1A—C8A	112.6 (2)	H8B1—C8B—H8B3	109.5
C2A—C1A—C6A	117.8 (3)	H8B2—C8B—H8B3	109.5
C2A—C1A—C7A	121.3 (2)	C3B—C9B—Br2B	113.29 (19)
C6A—C1A—C7A	120.9 (3)	C3B—C9B—H9B1	108.9
O3A—C6A—C5A	119.3 (2)	Br2B—C9B—H9B1	108.9
O3A—C6A—C1A	118.6 (3)	C3B—C9B—H9B2	108.9
C5A—C6A—C1A	122.0 (3)	Br2B—C9B—H9B2	108.9
C6A—C5A—C4A	118.1 (2)	H9B1—C9B—H9B2	107.7
C6A—C5A—C11A	120.7 (3)	O2B—C10B—H10G	109.5
C4A—C5A—C11A	121.0 (3)	O2B—C10B—H10H	109.5
O2A—C4A—C3A	118.1 (3)	H10G—C10B—H10H	109.5
O2A—C4A—C5A	120.2 (2)	O2B—C10B—H10I	109.5
C3A—C4A—C5A	121.6 (3)	H10G—C10B—H10I	109.5
C2A—C3A—C4A	118.3 (3)	H10H—C10B—H10I	109.5
C2A—C3A—C9A	120.9 (2)	C5B—C11B—Br3B	111.49 (18)
C4A—C3A—C9A	120.8 (3)	C5B—C11B—H11E	109.3
O1A—C2A—C1A	119.2 (2)	Br3B—C11B—H11E	109.3
O1A—C2A—C3A	118.6 (2)	C5B—C11B—H11F	109.3
C1A—C2A—C3A	122.1 (2)	Br3B—C11B—H11F	109.3
C1A—C7A—Br1A	113.43 (18)	H11E—C11B—H11F	108.0
C1A—C7A—H7A1	108.9	O3B—C12B—H12G	109.5
Br1A—C7A—H7A1	108.9	O3B—C12B—H12H	109.5
C1A—C7A—H7A2	108.9	H12G—C12B—H12H	109.5
Br1A—C7A—H7A2	108.9	O3B—C12B—H12I	109.5
H7A1—C7A—H7A2	107.7	H12G—C12B—H12I	109.5
O3A—C12A—H12D	109.5	H12H—C12B—H12I	109.5
O3A—C12A—H12E	109.5		
			
C8—O1—C2—C3	−99.1 (3)	O2A—C4A—C3A—C9A	5.3 (4)
C8—O1—C2—C1	85.4 (3)	C5A—C4A—C3A—C9A	−178.7 (2)
C6—C1—C2—O1	178.4 (2)	C8A—O1A—C2A—C1A	87.6 (3)
C7—C1—C2—O1	2.1 (4)	C8A—O1A—C2A—C3A	−95.8 (3)
C6—C1—C2—C3	3.1 (4)	C6A—C1A—C2A—O1A	179.9 (2)
C7—C1—C2—C3	−173.1 (2)	C7A—C1A—C2A—O1A	0.9 (4)
O1—C2—C3—C4	−177.8 (2)	C6A—C1A—C2A—C3A	3.4 (4)
C1—C2—C3—C4	−2.6 (4)	C7A—C1A—C2A—C3A	−175.6 (2)
O1—C2—C3—C9	−3.3 (4)	C4A—C3A—C2A—O1A	−179.8 (2)
C1—C2—C3—C9	172.0 (2)	C9A—C3A—C2A—O1A	−1.0 (4)
C10—O2—C4—C3	85.7 (3)	C4A—C3A—C2A—C1A	−3.3 (4)
C10—O2—C4—C5	−99.5 (3)	C9A—C3A—C2A—C1A	175.6 (2)
C2—C3—C4—O2	174.9 (2)	C2A—C1A—C7A—Br1A	−101.2 (3)
C9—C3—C4—O2	0.4 (4)	C6A—C1A—C7A—Br1A	79.8 (3)
C2—C3—C4—C5	0.2 (4)	C6A—C5A—C11A—Br3A	90.0 (3)
C9—C3—C4—C5	−174.3 (2)	C4A—C5A—C11A—Br3A	−94.2 (3)
O2—C4—C5—C6	−173.3 (2)	C2A—C3A—C9A—Br2A	101.2 (3)
C3—C4—C5—C6	1.5 (4)	C4A—C3A—C9A—Br2A	−80.0 (3)
O2—C4—C5—C11	2.8 (4)	C8B—O1B—C2B—C3B	−90.8 (3)
C3—C4—C5—C11	177.6 (2)	C8B—O1B—C2B—C1B	95.9 (3)
C12—O3—C6—C5	80.3 (3)	C6B—C1B—C2B—O1B	173.1 (2)
C12—O3—C6—C1	−104.2 (3)	C7B—C1B—C2B—O1B	−1.0 (4)
C4—C5—C6—O3	174.3 (2)	C6B—C1B—C2B—C3B	−0.1 (4)
C11—C5—C6—O3	−1.7 (4)	C7B—C1B—C2B—C3B	−174.2 (2)
C4—C5—C6—C1	−0.9 (4)	O1B—C2B—C3B—C4B	−173.0 (2)
C11—C5—C6—C1	−176.9 (2)	C1B—C2B—C3B—C4B	0.1 (4)
C2—C1—C6—O3	−176.7 (2)	O1B—C2B—C3B—C9B	−0.1 (4)
C7—C1—C6—O3	−0.5 (4)	C1B—C2B—C3B—C9B	173.0 (2)
C2—C1—C6—C5	−1.3 (4)	C10B—O2B—C4B—C5B	−100.6 (3)
C7—C1—C6—C5	174.9 (2)	C10B—O2B—C4B—C3B	86.7 (3)
C6—C1—C7—Br1	83.6 (3)	C2B—C3B—C4B—O2B	173.0 (2)
C2—C1—C7—Br1	−100.3 (3)	C9B—C3B—C4B—O2B	0.1 (4)
C2—C3—C9—Br2	88.5 (3)	C2B—C3B—C4B—C5B	0.5 (4)
C4—C3—C9—Br2	−97.1 (3)	C9B—C3B—C4B—C5B	−172.4 (2)
C6—C5—C11—Br3	−95.2 (3)	O2B—C4B—C5B—C6B	−173.8 (2)
C4—C5—C11—Br3	88.8 (3)	C3B—C4B—C5B—C6B	−1.1 (4)
C12A—O3A—C6A—C5A	−89.9 (3)	O2B—C4B—C5B—C11B	1.5 (4)
C12A—O3A—C6A—C1A	93.9 (3)	C3B—C4B—C5B—C11B	174.1 (2)
C2A—C1A—C6A—O3A	175.7 (2)	C12B—O3B—C6B—C5B	88.6 (3)
C7A—C1A—C6A—O3A	−5.4 (4)	C12B—O3B—C6B—C1B	−97.7 (3)
C2A—C1A—C6A—C5A	−0.4 (4)	C4B—C5B—C6B—O3B	174.6 (2)
C7A—C1A—C6A—C5A	178.6 (2)	C11B—C5B—C6B—O3B	−0.6 (4)
O3A—C6A—C5A—C4A	−178.6 (2)	C4B—C5B—C6B—C1B	1.2 (4)
C1A—C6A—C5A—C4A	−2.5 (4)	C11B—C5B—C6B—C1B	−174.1 (2)
O3A—C6A—C5A—C11A	−2.6 (4)	C2B—C1B—C6B—O3B	−174.0 (2)
C1A—C6A—C5A—C11A	173.4 (2)	C7B—C1B—C6B—O3B	0.1 (4)
C10A—O2A—C4A—C3A	−95.9 (3)	C2B—C1B—C6B—C5B	−0.6 (4)
C10A—O2A—C4A—C5A	88.1 (3)	C7B—C1B—C6B—C5B	173.6 (2)
C6A—C5A—C4A—O2A	178.6 (2)	C6B—C1B—C7B—Br1B	93.5 (3)
C11A—C5A—C4A—O2A	2.6 (4)	C2B—C1B—C7B—Br1B	−92.5 (3)
C6A—C5A—C4A—C3A	2.7 (4)	C2B—C3B—C9B—Br2B	93.7 (3)
C11A—C5A—C4A—C3A	−173.3 (2)	C4B—C3B—C9B—Br2B	−93.5 (3)
O2A—C4A—C3A—C2A	−175.8 (2)	C6B—C5B—C11B—Br3B	−97.1 (3)
C5A—C4A—C3A—C2A	0.1 (4)	C4B—C5B—C11B—Br3B	87.8 (3)

### Hydrogen-bond geometry (Å, º)

**Table:**

*D*—H···*A*	*D*—H	H···*A*	*D*···*A*	*D*—H···*A*
C8*A*—H8*A*1···Br1*A*	0.98	2.91	3.787 (3)	150
C8—H8*A*···Br1	0.98	2.84	3.702 (3)	148
C8—H8*C*···Br2	0.98	2.86	3.670 (3)	140
C7*B*—H7*B*2···Br3*B*^i^	0.99	2.92	3.665 (3)	133
C10—H10*A*···Br2	0.98	2.85	3.717 (3)	149
C10—H10*C*···Br3	0.98	2.89	3.673 (3)	138
C10*A*—H10*D*···Br3*A*	0.98	2.78	3.659 (4)	149
C10*B*—H10*G*···Br2*B*	0.98	2.72	3.606 (3)	151
C10*B*—H10*I*···Br3*B*	0.98	2.86	3.663 (3)	140
C11—H11*B*···O1*A*^ii^	0.99	2.55	3.429 (4)	148
C8*B*—H8*B*3···Br2*B*	0.98	2.85	3.697 (4)	146
C12*B*—H12*G*···Br3*B*	0.98	2.83	3.702 (3)	149

Symmetry codes: (i) *x*−1, *y*, *z*; (ii) −*x*+1, −*y*+1, −*z*.

## References

[bb1] Awwadi, F. F., Willett, R. D., Peterson, K. A. & Twamley, B. (2006). *Chem. Eur. J.* **12**, 8952–8960. 10.1002/chem.200600523 16972291

[bb2] Bruker (2008). *APEX2*, *SAINT* and *SADABS* Bruker AXS Inc., Madison, Wisconsin, USA.

[bb3] Desiraju, G. R. (2002). *Acc. Chem. Res.* **35**, 565–573. 10.1021/ar010054t 12118996

[bb4] Farrugia, L. J. (2012). *J. Appl. Cryst.* **45**, 849–854.

[bb5] Holec, J., Rybáček, J., Budešínsky, M., Pospíšil, L. & Holý, P. (2011). *Monatsh. Chem.* **142**, 821–826.

[bb6] Kim, J., Kim, Y., Park, N., Hahn, J. H. & Ahn, K. H. (2005). *J. Org. Chem.* **70**, 7087–7092. 10.1021/jo050654w 16122226

[bb7] Li, H., Homan, E. A., Lampkins, A. J., Ghiviriga, I. & Castellano, R. K. (2005). *Org. Lett.* **7**, 443–446. 10.1021/ol047597y 15673260

[bb8] Metrangolo, P. & Resnati, G. (2008). *Halogen Bonding: Fundamentals and Applications* pp. 181–191. Berlin: Springer.

[bb9] Sheldrick, G. M. (2008). *Acta Cryst.* A**64**, 112–122. 10.1107/S0108767307043930 18156677

[bb10] Simaan, S., Siegel, J. S. & Biali, S. (2003). *J. Org. Chem.* **68**, 3699–3701. 10.1021/jo034016u 12713382

[bb11] Steiner, T. (2002). *Angew. Chem. Int. Ed.* **41**, 48–76.

[bb12] Whiting, A. L. & Hof, F. (2012). *Org. Biomol. Chem.* **10**, 6885–6892. 10.1039/c2ob25882j 22828995

